# Mortality After Lower Extremity Amputation: A Portuguese Study

**DOI:** 10.7759/cureus.91199

**Published:** 2025-08-28

**Authors:** Maria Pires, João Diniz, Ana Aguiar, Marta Estrela, Pedro Cantista, Mário Vaz, Francisco Neves, Ana Fialho, Catarina Silva

**Affiliations:** 1 Physical Medicine and Rehabilitation, Unidade Local de Saúde do Algarve, Faro, PRT; 2 Physical Medicine and Rehabilitation, Unidade Local de Saúde Tâmega e Sousa, Penafiel, PRT; 3 Physical Medicine and Rehabilitation, Hospital Divino Espírito Santo, Ponta Delgada, PRT; 4 Medical Sciences, Universidade de Aveiro, Aveiro, PRT; 5 Physical Medicine and Rehabilitation, Unidade Local de Saúde de Santo António, Porto, PRT

**Keywords:** comorbidity, lower extremity amputation, mortality, portugal’s healthcare, rehabilitation, statins

## Abstract

Introduction

Despite being one of the earliest documented surgical procedures, lower extremity amputation (LEA) continues to be frequently performed in modern clinical practice. Currently, most LEAs are associated with diabetes mellitus (DM) and peripheral artery disease. Although long-term mortality after LEA remains high, recent evidence suggests that survival following major LEA may be improving. This trend is considered multifactorial, with advances in cardiovascular disease management being a major contributing factor. The aim of this retrospective study was to quantify the long-term mortality rate following LEA and to assess the relative contribution of comorbidities to overall mortality.

Methods

A single-center, observational, retrospective study was conducted at a central hospital in Portugal. Medical records of all patients who attended outpatient consultations in Physical Medicine and Rehabilitation - Amputees following LEA between January 2015 and June 2024 were analyzed.

Results

A total of 730 patients were included, with a mean age of 68 years; 72% were male. Nearly one-third of the patients had a history of smoking. During the follow-up period, 208 patients died, with a mean age at death of 71 years. The five- and 15-year mortality rates were 20% and 26%, respectively. The leading causes of amputation were vascular disease (64%) and trauma (19%). Amputations due to oncological disease were associated with the poorest survival outcomes, followed by those caused by vascular disease. With respect to amputation level, hip disarticulation was associated with the lowest long-term survival. Mortality rates were slightly higher for transfemoral amputations (31%) compared with transtibial amputations (28%). DM and hypertension were identified as risk factors for post-acute mortality.

Conclusions

This study confirms the high long-term mortality associated with LEA but also suggests that survival rates may be improving. Vascular disease remains the most common cause of LEA and the second leading contributor to poor survival outcomes. These findings highlight the importance of preventive strategies to reduce amputation rates and underscore the critical role of comprehensive, interdisciplinary care, including effective management of cardiovascular risk factors, in mitigating long-term mortality.

## Introduction

Hippocrates stated, “For extreme diseases, extreme methods of cure … are most suitable.” Amputation, one of the oldest surgical interventions, remains an aggressive but necessary treatment in modern healthcare [1]. Over time, its indications have evolved [2]. In the United States, advances in treatment modalities have led to a decline in lower extremity amputations (LEAs, defined as transtibial or more proximal amputations) related to traumatic events and cancer; however, those associated with diabetes mellitus (DM) and peripheral artery disease (PAD) continue to rise [3]. In Western countries, up to 75% of all LEAs are linked to these conditions [4].

Mortality rates after LEA remain notably high, with most studies reporting a five-year mortality ranging from 55% [5] to 70% [6]. However, a recent analysis of more than 25,000 patients reported a lower mortality rate of 18.1%, suggesting that survival following major LEA may be improving [7]. That cohort included perioperative deaths, which is important to consider when comparing mortality rates across studies. This trend is believed to be multifactorial, with advances in cardiovascular disease management being a contributing factor [7,8]. Cardiac disease, stroke, malignancy, renal complications, and DM are among the most common causes of death post-amputation, underscoring the role of comorbidities in patient outcomes [7].

The aim of this retrospective study was twofold: (1) to quantify long-term mortality following LEA at five and 15 years and (2) to evaluate the contribution of specific comorbidities, such as DM, hypertension, PAD, and dyslipidemia, along with patient habits such as alcohol use and smoking, as well as the etiology and level of amputation, to overall mortality. By addressing these factors, this study seeks to provide clinically relevant insights into prognosis and long-term care needs in this high-risk population.

## Materials and methods

Hospital de Santo António is a central hospital in Portugal that provides care and receives referrals from across the northern region of the country. A single-center, observational, retrospective study was conducted at this hospital, analyzing the medical records of all patients who attended outpatient consultations for Physical Medicine and Rehabilitation - Amputees between January 2015 and June 2024. Patients were excluded if they were being followed for upper limb amputation, had a congenital disorder, or if their medical records lacked relevant information. Of an initial cohort of 768 patients, 38 were excluded according to these criteria, resulting in a final sample of 730 patients. The sample included all consecutive patients followed at this hospital during the study period, ensuring representativeness of the population under care. Observations with missing data or lost to follow-up were excluded from the analyses.

Demographic characteristics, comorbidities (DM, hypertension, PAD, and dyslipidemia), patient habits (alcohol use and smoking), and long-term mortality data were extracted from the medical records of outpatient consultations reviewed for the purposes of this study. Long-term mortality data were also confirmed through these records.

All statistical analyses were performed using R software (v. 4.4.1), with the packages readxl, dplyr, car, survival, and survminer. Descriptive statistics were calculated for the variables of interest. Independent samples t-tests were conducted to assess differences in age at death and time after amputation across binary comorbidity variables, with statistical significance set at p < 0.05. For categorical variables, ANOVA was employed (p < 0.05). Multivariable logistic regression was performed to determine the association between comorbidities and mortality, with ORs and 95% CIs calculated to quantify the strength of associations. Variance inflation factors were computed for all variables (range: 1.09-1.70), indicating no evidence of problematic multicollinearity; therefore, no variables were excluded from the model. Survival analysis was conducted using Kaplan-Meier survival curves to estimate survival probabilities after amputation, with log-rank tests applied to compare survival distributions across groups (p < 0.05).

## Results

A total of 730 patients were included in this study, with a mean age of 68 years (range: 26-101). Of these, 72% were male. Nearly one-third of the patients (34%) had a history of smoking, with a significantly higher prevalence among men than women (42% vs. 12%). During the follow-up period, 208 patients died, with a mean age at death of 71 years. The five- and 15-year mortality rates were 20% and 26%, respectively.

**Figure 1:**
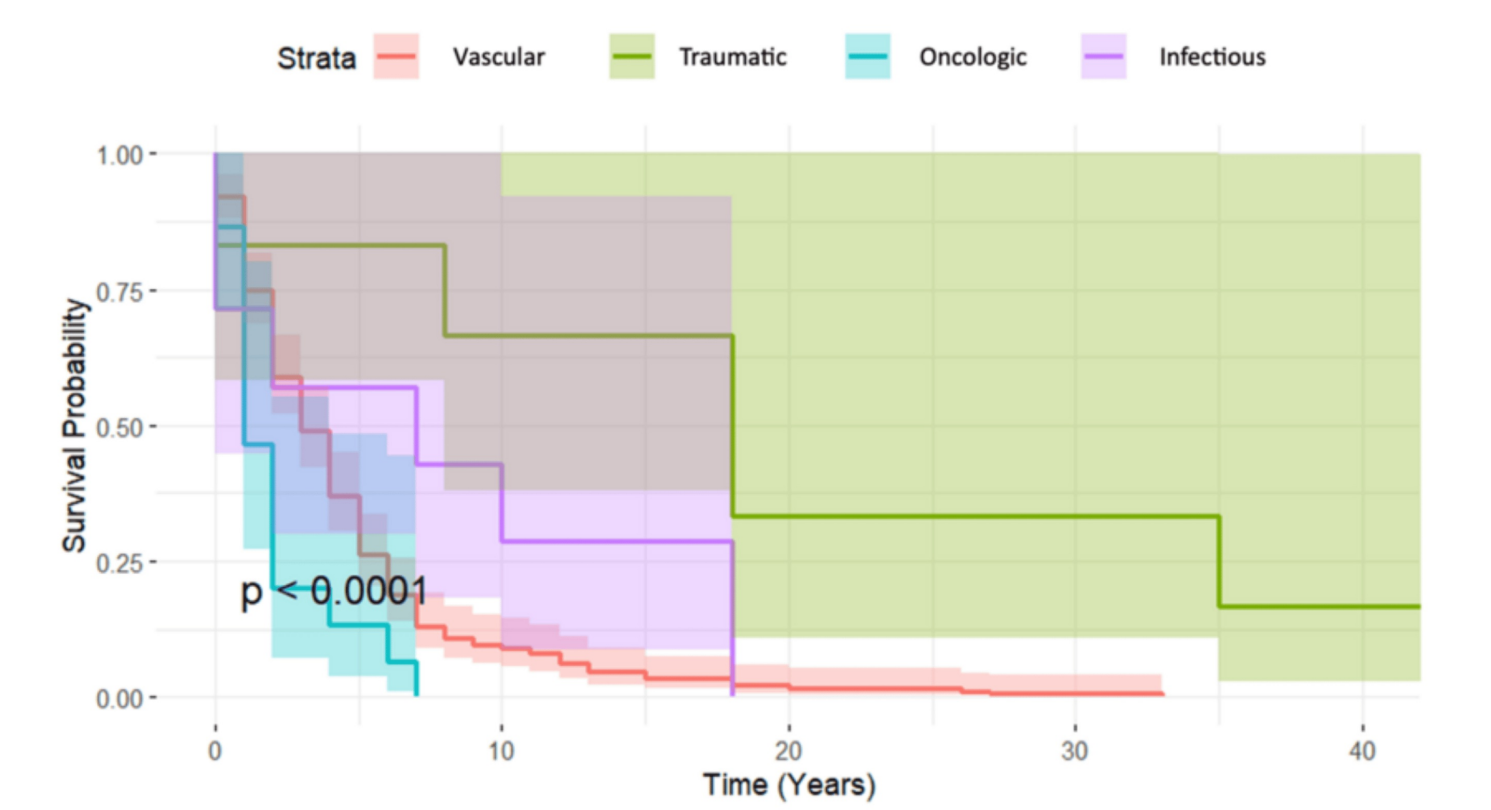
Kaplan-Meier survival curves by amputation etiology Survival distributions were compared using the log-rank test (p < 0.05). Rare causes (e.g., CREST syndrome) were excluded from the analysis.

The most common amputation levels were transtibial (351 patients) and transfemoral (316 patients). The primary causes of amputation were vascular (64%), traumatic (19%), oncologic (7%), and infectious (5%). Significant differences in age at death were observed across the different amputation etiologies (ANOVA test, p < 0.05). Mortality outcomes also varied significantly according to amputation etiology and level. Kaplan-Meier analysis showed that amputations due to oncologic disease were associated with the poorest survival outcomes, followed by those caused by vascular disease (log-rank test, p < 0.05; Figure 1).

**Figure 2:**
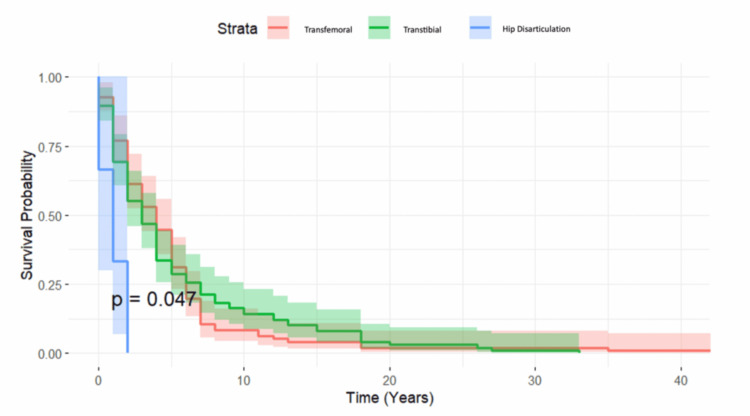
Kaplan-Meier survival curves by amputation level Survival distributions were compared using the log-rank test (p < 0.05).

Regarding amputation level (Figure 2), hip disarticulation was associated with the poorest long-term survival. In this cohort, seven patients underwent this procedure, six of whom had oncologic disease. Mortality rates were slightly higher for transfemoral amputations (31%) compared with transtibial amputations (28%). 

Multivariable logistic regression (Table 1) identified DM and hypertension as significant risk factors for post-acute mortality (p < 0.05), whereas dyslipidemia under statin treatment was found to be a protective factor (p < 0.05). A t-test analysis of comorbidities revealed significant differences in mean age at death among patients with hypertension, tobacco use, and alcohol consumption (p < 0.05 for all).

**Table 1:** Multivariate regression results Amp., amputation; DM, diabetes mellitus; HTN, hypertension; PAD, peripheral artery disease

Variable	OR	CI (lower)	CI (upper)	p-Value
DM	1.733	1.033	2.953	0.040
HTN	3.399	1.619	7.530	0.002
Dyslipidemia	0.497	0.262	0.944	0.032
PAD	0.947	0.568	1.590	0.836
Tobacco	1.122	0.663	1.890	0.664
Alcohol	1.723	0.878	3.321	0.107
Sex (male/female)	0.661	0.390	1.130	0.127
Age (years)	1.007	0.989	1.026	0.422
Amp. level	0.851	0.551	1.310	0.464
Amp. cause	0.949	0.688	1.283	0.742

## Discussion

To our knowledge, this is the first study to report overall mortality in amputees in Portugal. More than two-thirds of the patients were male, reflecting the higher incidence of amputations among men, likely influenced by lifestyle factors such as smoking (42% in men vs. 12% in women). According to the National Institute of Statistics, 17.0% of the Portuguese population aged 15 years and older were smokers in 2019, with a male-to-female ratio of 2:1 [9]. This predominance of men should be considered when generalizing our findings, as outcomes may differ in populations with a more balanced sex distribution.

Over the nine-year study period, 208 patients died, with five- and 15-year mortality rates of 20% and 26%, respectively. Most studies have reported a five-year mortality following LEA of 55% [5] to 70% [6], as supported by a 2017 meta-analysis [10]. However, a recent study of more than 25,000 patients reported a much lower mortality of 18.1% [7]. This discrepancy may be explained by several factors: many earlier studies recruited cohorts in the early 2000s, when PAD was undertreated, particularly with regard to statin therapy. In addition, advances in wound care have likely helped prevent higher-level amputations [7].

The relatively low mortality observed in our cohort should be interpreted in the context of the exclusion of perioperative deaths, which would have increased overall mortality, as well as the benefits of the comprehensive, specialized care provided at our high-volume physical medicine and rehabilitation (PMR) center. At Hospital de Santo António, all patients undergoing amputation surgery are evaluated by a PMR physician before discharge, with follow-up consultations scheduled as part of routine care. The hospital’s Diabetic Foot Group meets weekly to assess hospitalized patients with neuropathic or neuroischemic diabetic foot ulcers and determine the most appropriate therapeutic strategies. Additionally, the hospital provides a specialized outpatient diabetic foot consultation, where patients are assessed for cutaneous and neurovascular integrity. Therapeutic interventions include callus debridement, ulcer treatment, and correction of nail abnormalities. Recent evidence also highlights the effectiveness of preventive strategies such as rocker-bottom shoes and orthotic insoles in redistributing plantar pressure and reducing complications in diabetic patients [11]. This approach is supported by an interdisciplinary team, including vascular and orthopedic surgeons, endocrinologists, podiatrists, and physical therapists, ensuring coordinated and holistic patient management.

Kaplan-Meier analysis (Figure 1) showed that amputations due to oncologic disease had the worst prognosis, with hip disarticulations, frequently associated with oncologic disease, consistently showing the poorest outcomes (Figure 2). Vascular disease followed as the second leading etiology associated with the lowest survival outcomes and accounted for 64% of LEAs in our cohort, indicating its significant impact on mortality. Given the preventable nature of many cardiovascular diseases, adopting heart-healthy behaviors, such as a balanced diet, regular physical activity, smoking cessation, and effective risk factor management, could greatly reduce this burden [12].

Regarding risk factors for increased mortality, DM and hypertension were identified as statistically significant contributors. Interestingly, a diagnosis of dyslipidemia was found to be a protective factor. Robust evidence supports statin therapy as an effective intervention for reducing cardiovascular mortality risk [12]. A prospective study by DeCarlo et al., involving 811 patients who underwent major LEA, demonstrated that medium- and high-intensity statin therapy was associated with a survival benefit at one year [8]. We acknowledge that dyslipidemia is often linked to statin initiation, which raises the possibility of confounding bias: the observed protective association may reflect the effects of treatment rather than the diagnosis itself. Treatment data (e.g., prescription or adherence to statins) were not available, so this finding should be interpreted with caution. Hypertension, tobacco use, and alcohol consumption were also shown to influence the mean age at death, underscoring the importance of monitoring these risk factors in amputee patients during follow-up.

In line with existing literature, above-knee amputation (AKA) was associated with a slightly higher mortality rate than below-knee amputation, 31% and 28%, respectively. This difference is likely related to the greater functional limitations associated with AKA, which result in reduced mobility and, consequently, poorer cardiovascular health [13]. Another factor to consider is the potentially more advanced vascular disease at the time of amputation in AKA patients [7].

This study has several limitations. Data were collected from post-discharge PMR consultations, which did not capture perioperative or short-term mortality. As some patients may not have survived long enough to attend these consultations, they were excluded. This selection bias may have led to an underestimation of overall mortality and could also have influenced the observed predictors of mortality, as patients with higher perioperative risk were not represented. Furthermore, because the study relied on medical records, some comorbidities may not have been fully documented, and certain potentially relevant conditions, such as ischemic heart disease, were not included. Finally, the study was conducted at a single medical center, which may limit the generalizability of the findings.

## Conclusions

This study highlights the high long-term mortality associated with LEAs, although survival rates appear to be improving, likely due to advances in medical management and rehabilitation. Mortality was strongly influenced by comorbidities, with vascular disease being the leading cause of amputation and the second most significant factor associated with reduced survival. The observed protective effect of dyslipidemia may reflect the mortality benefit of statin therapy, as supported by previous literature. Future research directly assessing statin use in amputee populations would help clarify this association.

Our findings also suggest that specialized, interdisciplinary care, such as that provided at our high-volume PMR center, can contribute to improved outcomes. Structured follow-up with an interdisciplinary team, including vascular and orthopedic surgeons, endocrinologists, podiatrists, and physical therapists, may reduce complications, enhance functional recovery, and ultimately improve long-term survival. These results also reinforce the value of preventive strategies to reduce amputation rates, including effective management of cardiovascular risk factors, in mitigating long-term mortality. Despite its retrospective design and the exclusion of perioperative and short-term mortality, this study provides valuable insights into long-term survival after LEA.
